# Etiology and recovery of knee extensor muscle fatigue following simulated basketball match-play

**DOI:** 10.5114/biolsport.2025.148546

**Published:** 2025-04-01

**Authors:** Davide Ferioli, Tomás T. Freitas, Carmen Mannucci, Linda Chung, Andrea Mombelli, Pedro E. Alcaraz, Nicola A. Maffiuletti

**Affiliations:** 1 Department of Biomedical Sciences, Dental Sciences, and Morpho-Functional Imaging, University of Messina, Messina, Italy; 2 UCAM Research Center for High Performance Sport, UCAM Universidad Católica de Murcia, Murcia, Spain; 3 Facultad de Deporte, UCAM Universidad Católica de Murcia, Murcia, Spain; 4 SCS – Strength and Conditioning Society, Murcia, Spain; 5 NAR – Nucleus of High Performance in Sport, São Paulo, Brazil; 6 Human Performance Lab, Schulthess Clinic, Zurich, Switzerland

**Keywords:** Basketball Activity Simulation, Protocol, Contractility, Fatigability, Maximal Voluntary Contraction, Simulated Game, Team Sport

## Abstract

This study investigated the etiology and recovery of knee extensor muscle fatigue following simulated basketball match-play. Thirteen adult male competitive basketball players (age: 25 ± 4 years, stature: 185 ± 9 cm, body mass: 86 ± 14 kg, body fat: 13 ± 4%) completed a simulated match-play (i.e., the Basketball Activity Simulation Protocol) consisting of standardized specific match-based basketball activities. Before (PRE) and immediately after (POST) the match-play, the neuromuscular function of the knee extensors was evaluated to determine the amount of muscle fatigue and its origin. Assessments were also repeated 24 h (POST24) and 48 h (POST48) after the match-play to evaluate muscle fatigue recovery. The main outcomes were maximal voluntary contraction (MVC) torque, voluntary activation estimated through superimposed stimuli, electrically-evoked twitch and doublet peak torque (PT), and the 10:100 Hz doublet ratio. The Total Quality Recovery (TQR) scale was used to assess the perceived recovery status at PRE, POST24 and POST48. Time-related changes (oneway repeated-measures ANOVA) were observed for MVC torque (main effect: P = 0.002, moderate; post hoc: POST < PRE, small), twitch and doublet PT (P < 0.001, strong; POST < PRE, POST24 and POST48, moderate-to-large) and 10:100 Hz doublet ratio (P < 0.001, strong; POST < PRE, POST24 and POST48, large; POST48 > PRE, moderate). Voluntary activation and TQR were not affected at the different time-points (P = 0.060 and P = 0.455, minimum, respectively). In conclusion, basketball match-play significantly reduced knee extensor MVC strength, with baseline levels being restored within 24 h. Muscle fatigue was accompanied by a significant pre-to-post match reduction of electrically-evoked torque responses, indicative of peripheral fatigue (and evidence of low-frequency fatigue), while no signs of central fatigue were noted.

## INTRODUCTION

Basketball is an intermittent team sport encompassing high- and low-intensity playing phases that place significant physical demands on players [1, 2]. Basketball players are indeed frequently required to perform high-intensity accelerations, decelerations, changes of direction, sprints and jumps over the course of the match [2, 3]. Because of the repetition of these efforts, players may experience a reduction of physical performance across the match [4, 5], which could potentially affect the match outcome. Accordingly, high-intensity activities and high-speed running were reported to decrease across basketball matches, with the most notable declines occurring between the first and fourth quarters [4, 5]. In line with these findings, most of the studies on the topic reported sprint, repeated sprint, and jump performance to be reduced from pre- to post-match across various player samples [6]. These detrimental effects may also persist in the days following a match [6], with the associated recovery being therefore a key factor for success during match-congested periods like playoffs [7]. Accordingly, significant impairments in sprint and jump performance were reported up to 48 hours after basketball activity [6], while repeated sprint ability was found to be restored within 24 hours after a match [6].

Contrary to the numerous investigations on the effect of basketball match-play on physical performance (e.g., jumping and sprinting), only a limited number of studies has focused on knee extensor muscles that are heavily involved in key physical tasks like jumping, sprinting and changing directions [8–10]. This is likewise related to the concept of muscle fatigue, which may originate from central and/or peripheral factors. Specifically, the assessment of isometric knee extension torque during voluntary, electrically-evoked (through transcutaneous electrical nerve/muscle stimulation) and combined contractions before vs after a fatiguing task allows us to characterize the amount of muscle fatigue (defined as the decline in maximal voluntary torque), central fatigue (defined as the decline of voluntary activation, VA) and peripheral fatigue (defined as the decline of evoked torque) [11, 12]. These measurements have been used for decades to characterize the amount, etiology and recovery of exercise-induced fatigue in athletes from different sports, with the ultimate goal of understanding and potentially counteracting the impact of central and peripheral fatigue on sport performance [11–13]. In basketball, Delextrat et al. [10] found a significant decrease in the isokinetic strength of knee flexors but not of knee extensors after an official match in professional female basketball players. Scanlan et al. [9] showed *trivial-to-small* variations in isokinetic knee extensor and flexor strength after simulated basketball match-play (i.e., 4 × 10-min quarters of the Basketball Exercise Simulation Test) in young male players. Finally, Ansdell and Dekerle [8] found isometric knee extensor strength to be reduced by 15% after simulated basketball match-play (i.e., the modified Loughborough Intermittent Sprint Test) in adult male players. In addition, these authors delivered various electrical stimuli to the femoral nerve under resting conditions to examine peripheral fatigue induced by basketball and revealed the occurrence of both high- and low-frequency fatigue. Taken together, these studies provide relevant information about the impact of basketball match-play on knee extensor muscle function, but more research is needed about the central and peripheral mechanisms contributing to fatigue development and its recovery process. This information is crucial for basketball practitioners both for implementing appropriate recovery strategies and for developing effective training programs, with the ultimate goal of optimizing player readiness during match-congested periods. From a practical point of view, knowing the time course of central and peripheral fatigue would help tailor a recovery intervention to the specific needs of a player. For example, in the case of a predominant occurrence of peripheral fatigue, dietary supplementation could be considered as an adjunct to the classical recovery modalities [14]. On the other hand, in case of a predominant occurrence of central fatigue, active recovery modalities may be preferred to passive interventions [15].

When evaluating muscle fatigue in team sports, a crucial step is the selection of the most appropriate match-play protocol as it can substantially impact the study outcomes. While collecting neuromuscular data during official matches would be the best approach in terms of ecological validity, this type of studies is challenging due to coaching staff restrictions and players’ availability. Furthermore, unlike other team sports like soccer, in basketball there are no pre-determined substitutions and only five players per team finish the match on the court, while the others sit on the bench. Thus, it is almost impossible to quantify post-match fatigue for all team players. In addition, playing time is a main confounding factor, as it may markedly influence the amount and type of fatigue. Thus, simulation match-play protocols have been applied for this type of research in team sports [16], including basketball [8, 9]. However, it should be considered that the simulated match-play protocols previously adopted in basketball [8, 9] did not perfectly reflect the actual playing time (i.e., players are not used to compete for the entire 40-min match), physical activities (e.g., lacking lateral shuffle and multidirectional changes of direction) and breaks (e.g., not encompassing free throws, time-outs, substitutions) typically encountered during official matches. In this regard, the basketball activity simulation protocol (BASP) was recently developed to specifically replicate actual playing durations and match configurations, holding strong translation to real competitive contexts, and with reliability and discriminant validity being reported [17]. Thus, the BASP may represent a valid alternative to official matches for a preliminary exploration of muscle fatigue induced by basketball match-play.

Therefore, the aim of the present study was to investigate the etiology and recovery of knee extensor muscle fatigue following a simulated basketball match-play (i.e., the BASP) in adult male competitive players.

## MATERIALS AND METHODS

### Subjects

Thirteen trained (tier 2) [18] adult male basketball players (age: 25 ± 4 years, stature: 185 ± 9 cm, body mass: 86 ± 14 kg, body fat: 13 ± 4%) who were competing in the Spanish 5^th^ division (Campeonato de España de Primera División Nacional) were recruited for this study. During the regular season, they completed at least three on-court team training sessions (each lasting ~90–120 min) and a game per week. All players were over 18 years of age, were free from any injury across the study and had more than 5 years of competitive basketball experience. They volunteered to participate after being informed of the study procedures, risks, and benefits. All procedures were approved by the UCAM Universidad Católica de Murcia’s Ethics Committee (CE052207) with written informed consent obtained from each player prior to participation.

### Design

**FIG. 1:**
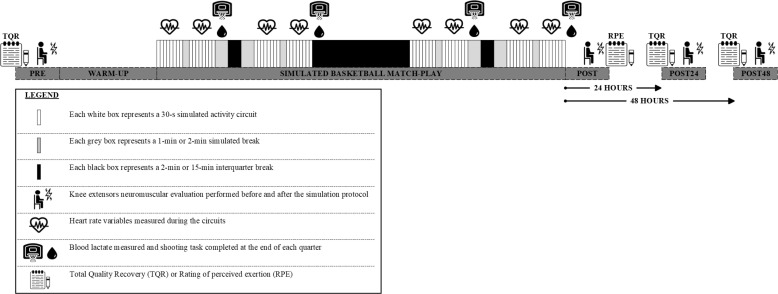
Graphical representation of the study design including the configuration of the simulated basketball match-play protocol.

The study was performed in June and July 2022, within two months of finishing the 2021–2022 competitive season (May 2022). All players were familiarized with the testing procedures before data collection, which took place over three consecutive days. On day one, players completed the BASP consisting of standardized specific match-based basketball activities [17]. Before (PRE) and immediately after (POST) the BASP, neuromuscular evaluations were performed to quantify muscle fatigue and its underlying origin [13]. Assessments were also repeated 24 hours (POST24) and 48 hours (POST48) after the BASP to examine the recovery of muscle fatigue (Figure 1). During the entire testing period, players were instructed to maintain their regular eating and sleeping behaviors and to abstain from a physical activity from 24 hours before the first testing day. Their perceived recovery status was monitored on a daily basis by means of the Total Quality Recovery (TQR) scale. All testing sessions were performed on the same indoor basketball court in a controlled air-conditioned environment following standardized procedures.

### Experimental procedures

#### Simulated Basketball Match-play

**FIG. 2:**
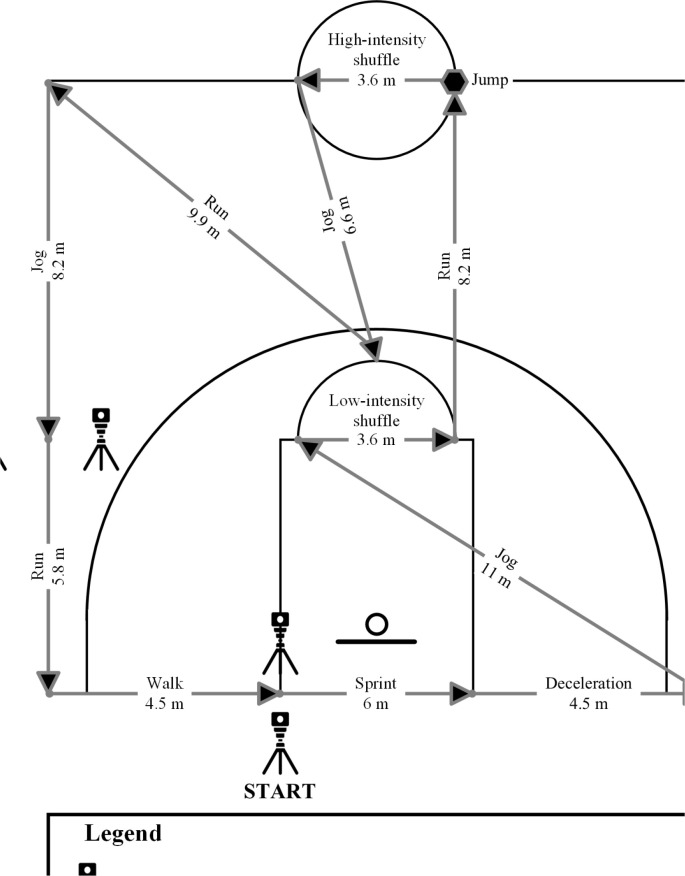
The activity breakdown within each circuit of the simulated basketball match-play (recreated from the original work of Ferioli et al. [17]).

Players completed a standardized 15-min warm-up consisting of 5 min of active mobility exercises, 5 min of running skills and 5 min of basketball-specific skill exercises (ten 2-point shots, ten 3-point shots, and ten free throws). Thereafter, they underwent familiarization (i.e., they received verbal instructions and completed two circuit trials) before completing the simulated basketball match-play. According to the original version of the BASP [17], the simulated match-play lasted a total of 63 min with 32 min of activity being performed, representing live active play. The BASP was split into quarters, with each quarter involving two 4-min activity bouts separated by 1 min of passive seated rest (corresponding to a timeout duration), and 2 min of passive seated rest (corresponding to a substitution). Each quarter was further separated by 2 min of passive seated rest, with 15 min of passive seated rest between the second and third quarters (i.e., half-time break) in line with international regulations (Figure 1). Each 4-min simulated activity bout consisted of eight 30-s circuits that were arranged identically to the Basketball Exercise Simulation Protocol [19]. Each activity of the simulated basketball match-play was described to players before testing to guide movement intensity according to the original version of the BASP [17]. The activity breakdown within each circuit of the simulated basketball match-play is displayed in Figure 2.

To guarantee that timing was not initiated from a rolling start, the players started each circuit in a stationary position 30 cm behind the initial set of timing lights (Witty System; Microgate; Bolzano, Italy) via floor markings. Each circuit lasted for 30 s (maximum of 16 circuits completed per 8 min of activity per quarter). If players completed the circuit in less than 30 s, the remaining time was used as a passive standing rest at the starting point. If players completed the circuit in more than 30 s, they were required to completely stop then immediately commence the following circuit. In these cases, players completed less than 8 circuits per 4-min bout unless adequate timing was restored (i.e., they were completing circuits within 30 s when averaged across the 4-min bout). If players completed all allotted circuits across all quarters, they covered a total distance of 4519.2 m (564.9 m per 4-min bout consisting of 7 circuits of 71.9 m each and 1 circuit of 61.6 m). Players were given standardized verbal instructions and encouragement to ensure correct execution and optimal performance throughout each circuit.

A range of variables was collected for each player and tabulated across the entire BASP. Heart rate (HR) signal was continuously monitored using Firstbeat Sports sensors (Firstbeat Technologies Oy; Jyväs-kylä, Finland), which have supported reliability and validity [20]. Mean and peak HR values were determined through the BASP (i.e., excluding breaks) in absolute terms (bpm) and relative to the age-predicted maximum HR (i.e., 220 – age in years). At the end of each quarter, blood lactate concentration (Blact) was measured from the earlobe immediately after the completion of the last circuit using a portable amperometric microvolume lactate analyzer with supported validity and reliability [21] (Lactate Pro 2, Arkray, Kyoto, Japan). The average Blact determined across all quarters was reported. Rating of perceived exertion (RPE) was collected 30 min after the completion of the BASP using the CR-10 Borg’s scale [22] that is widely used in basketball research [7, 23, 24]. Also, players performed a shooting task for technical assessment (i.e., scoring the maximum number of free throws out of 25 attempts) following psychobiological evaluations (i.e., Blact and RPE) at the end of each quarter [25]. This was normally completed within 1 min not to take up a considerable portion of end-of-quarter breaks.

### Knee extensors neuromuscular evaluations

**FIG. 3:**
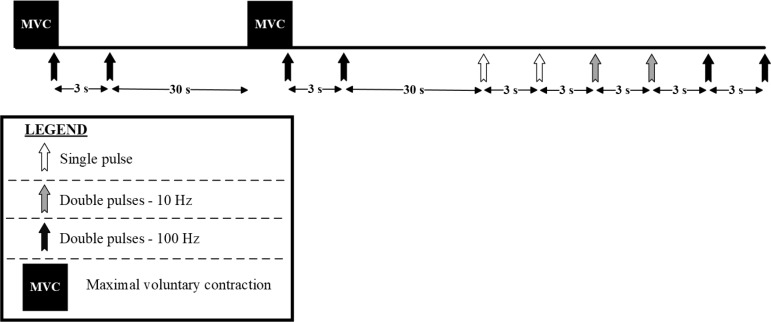
Overview of the neuromuscular evaluations.

All neuromuscular evaluations were performed on the right knee extensor muscles with the players seated on an isokinetic dynamometer (Biodex system 3, Shirley, NY, USA). The knee and hip angles were fixed at 90° (0° = knee fully extended), with Velcro® straps positioned across the chest, hip and thigh to minimize extraneous movements. Measurements included maximal voluntary contraction (MVC) torque, VA level, and electrically-evoked torque (Figure 3).

First, players completed a standardized warm-up consisting of three 4-s submaximal voluntary contractions at ~25, 50 and 75% MVC, separated by 20 s of rest. Second, stimulation intensity of a single rectangular pulse (1-ms duration) was progressively increased (10-mA increments) until a plateau in twitch peak torque (PT) was observed. This intensity was further increased by 20% to ensure maximal recruitment and then it was maintained for the ensemble of the stimuli [26, 27]. This procedure was completed at PRE, POST24 and POST48, but not at POST as the same stimulation intensity of PRE was used. Measurements consisted of (1) two 5-s MVCs with superimposed paired pulses (inter-pulse interval: 10 ms) – to evoke a superimposed doublet, followed by resting paired pulses (inter-pulse interval: 10 ms) ~3 s after the MVC – to evoke a 100-Hz potentiated doublet, (2) two single pulses at rest – to evoke a twitch response, (3) two paired pulses (inter-pulse interval: 100 ms) at rest – to evoke a 10-Hz doublet, and (4) two paired pulses (inter-pulse interval: 10 ms) at rest – to evoke a 100-Hz doublet, with 3 s of rest between all stimuli (Figure 3). Strong verbal encouragement was provided during the MVCs [12, 28], while participants were instructed to stay still and relaxed during the series of stimuli at rest. Electrically-evoked contractions were obtained by stimulating the femoral nerve with a constant-current high-voltage stimulator (Digitimer DS7R, Hertfordshire, United Kingdom). Surface self-adhesive electrodes (Tens, Macon, France) were placed in the femoral triangle (cathode, 5 × 5 cm) and in the gluteal fold (anode, 10 × 5 cm). Electrodes were positioned by the same examiner and their location was marked on the skin for repeated assessments (participants were required to keep these marks for the duration of the study).

Knee extensor torque-time data were recorded at 100 Hz, digitally filtered (IIR low pass filter at 10 Hz) [29] and analyzed using the AcqKnowledge software (version 3.9.1, Biopac Systems, Inc., Santa Barbara, CA). MVC torque was retained as the highest single value recorded during the 5-s contraction. The level of VA was estimated according to the twitch interpolation technique [30], where:

VA (%) = [(1 – superimposed doublet PT / 100-Hz potentiated doublet PT) × 100].

The PT of all twitch and doublet responses was quantified and the ratio between the 10-Hz doublet PT and the 100-Hz doublet PT was also calculated (10:100 Hz ratio) and considered as an index of low-frequency fatigue [31]. For the ensemble of these variables, the mean value of the two trials conducted in each condition was systematically retained.

### Total Quality Recovery

Players’ perceived recovery status was monitored at PRE, POST24 and POST48 (before the commencement of neuromuscular evaluations) using the TQR scale [32]. This scale has been widely applied in basketball research [33, 34] and has been shown to be an appropriate index of recovery state in team sport players [35].

### Statistical analysis

Descriptive results are reported as mean values ± SD. For each variable, the assumption of normality was verified with the Kolmogorov-Smirnov test. A series of one-way repeated-measures ANOVA was then utilized to assess time-related changes. Depending on the variable, the time factor included three (PRE, POST24 and POST48) or four (PRE, POST, POST24 and POST48) levels. Partial eta-squared (ƞ^2^) was used as a measure of effect size (ES) and respective values were classified as follows: < 0.04, *no effect*; 0.04–0.24, *minimum effect*; 0.25–0.63, *moderate effect*; ≥ 0.64, *strong effect* [36]. When significant F values were found, Bonferroni post hoc tests were used and Cohen’s d ES [37] were calculated. ES were classified as follows: < 0.20, *trivial*; 0.20–0.59, *small*; 0.60–1.19, *moderate*; 1.20–1.99, *large*; and 2.00–4.00, *very large* [38]. Statistical significance was set at *P* < 0.05. The SPSS software (version 20.0, IBM SPSS Statistics, Chicago, IL, USA) was utilized for all analyses.

## RESULTS

During the simulated basketball match-play, the players covered a total distance of 4479 ± 93 m. The mean and peak HR recorded during the protocol were 180 ± 5 bpm (corresponding to 92 ± 2% of HR max) and 192 ± 5 bpm (98 ± 2% of HR max), respectively, while the average Blact recorded during the BASP was 9.1 ± 2.6 mmol · L^−1^. The RPE collected at the end of the BASP was 7.1 ± 0.9 (very hard).

Regarding knee extensor neuromuscular fatigue, the main results are presented in Table 1. A main effect of time was observed for MVC torque (*P* = 0.002, *moderate*), with post hoc analyses revealing lower values at POST compared to PRE (*P* = 0.004, *small*). On the contrary, VA was not affected at the different time points (*P* = 0.060, *minimum*). A main effect of time was observed for all the electrically-evoked responses (all *P* < 0.001; *strong*). Post hoc analyses revealed that all twitch and doublet PT values were lower at POST compared to the other time points (all *P* ≤ 0.001, *moderate-to-large*). Similarly, the 10:100 Hz ratio was lower at POST compared to PRE, POST24 and POST48 (all *P* < 0.001, *large*). Furthermore, the 10:100 Hz ratio was lower at PRE than at POST48 (*P* = 0.002, *moderate*).

**TABLE 1:** Knee extensor neuromuscular function and perceived recovery before and after basketball match-play.

Variable	PRE	POST	POST24	POST48	ANOVA P value	Effect size – η^2^ (descriptor)
MVC torque (N · m)	313 ± 60	286 ± 69*	303 ± 76	303 ± 71	**0.002**	0.33 (*moderate*)
VA (%)	92.9 ± 5.5	90.9 ± 6.0	89.8 ± 6.9	88.2 ± 7.8	0.060	0.18 (*minimum*)
Twitch PT (N · m)	70.6 ± 10.7	49.8 ± 10.7*^#†^	72.9 ± 9.1	71.7 ± 12.1	**< 0.001**	0.83 (*strong*)
10-Hz doublet PT (N · m)	112.8 ± 18.0	66.2 ± 16.5*^#†^	115.6 ± 16.6	117.3 ± 18.7	**< 0.001**	0.90 (*strong*)
100-Hz doublet PT (N · m)	116.0 ± 20.6	93.1 ± 18.9*^#†^	117.5 ± 19.9	114.7 ± 23.0	**< 0.001**	0.71 (*strong*)
10:100 Hz ratio	0.98 ± 0.08^†^	0.71 ± 0.11*^#†^	0.99 ± 0.13	1.03 ± 0.10	**< 0.001**	0.82 (*strong*)
TQR (0–20)	16.4 ± 1.6	–	15.6 ± 2.5	16.3 ± 2.0	0.455	0.06 (*minimum*)

Data are mean ± SD. Abbreviations: MVC, maximal voluntary contraction; POST, immediately after the simulated basketball match-play; POST24, 24 hours after the simulated basketball match-play; POST48, 48 hours after the simulated basketball match-play; PRE, before the simulated basketball match-play; PT, peak torque; VA (%), voluntary activation. Notes: Bold *P* values indicate significant differences at *P* < 0.05;

* significantly different from PRE at *P* < 0.05;

# significantly different from POST24 at *P* < 0.05;

† significantly different from POST48 at *P* < 0.05.

The repeated ANOVA revealed no significant time effect (F_(2,24)_ = 0.814, *P* = 0.455, ƞ^2^ = 0.064) for TQR (Table 1).

## DISCUSSION

The present study is the first investigating the etiology and recovery of knee extensor muscle fatigue after a simulated basketball matchplay in trained adult male players. Basketball match-play significantly reduced knee extensor MVC strength immediately after match-play simulation, with baseline levels being restored within 24 hours. Such muscle fatigue was accompanied by a significant pre-to-post match reduction of electrically-evoked torque responses, indicative of peripheral fatigue, while no signs of central fatigue were noted. Peripheral fatigue affected more the torque evoked at low than high stimulation frequencies (low-frequency fatigue), but these alterations lasted less than 24 h.

We observed a significant decline of MVC strength immediately after the simulated match-play, confirming the occurrence of knee extensor muscle fatigue in agreement with the study of Ansdell and Dekerle [8]. The pre-to-post match reduction observed here (~9%, *small* ES) is slightly lower compared to the ~15% reported by Ansdell and Dekerle [8] probably due to inter-study differences in the simulated basketball match-play protocol (our protocol included more breaks to better reflect the active-rest phases of actual matchplay [17]). The MVC strength loss – rated as *small* – should be considered by practitioners as it may lead to a reduction of players’ physical performance (e.g., altering sprinting mechanisms and speed production) and consequently affect the match outcome [8].

Regardless of the amount of muscle fatigue (i.e., the exercise-induced reduction in the ability of a muscle to generate force), understanding its related mechanisms (i.e., central vs peripheral factors) may have important implications for practitioners, from a recovery perspective [39]. The present study is the first investigating potential changes in quadriceps VA induced by basketball match-play. No significant reductions in VA were observed therefore indicating the absence of central fatigue after match-play. This could be explained by the relatively short match-play (63 min) and playing time (32 min) of our protocol, as in fact significant quadriceps VA reductions (~7%) were observed following longer-duration real and simulated matchplay in other team sports such as soccer [16, 40]. Contrary to VA, all the indicators of peripheral fatigue (evoked peak torque of twitch and doublet responses) were significantly impaired after our simulated basketball match-play. These pre-to-post reductions ranged from 20% (100-Hz doublet) to 43% (10-Hz doublet) with *moderate-to-large* ES, thus highlighting the impact of this type of fatigue. Unsurprisingly, these changes were approximately twofold compared to those reported by Ansdell and Dekerle [8] for similar evoked responses. Their protocol, which entailed several linear activities of 15–20 m (e.g., walk, jog, run), few explosive actions such as sprints and no lateral shuffles, accelerations and decelerations over short distances, was certainly less demanding and less representative of real basketball match-play compared to our BASP. This could explain the inter-study differences in the amount of peripheral fatigue as well as the *large* reduction in the 10:100 Hz ratio we observed (contrary to Ansdell and Dekerle [8]), that is indicative of high levels of low-frequency fatigue. This form of peripheral fatigue, which may reflect a decrease in calcium release and/or sensitivity influencing the excitation-contraction coupling [41], is frequent following repeated explosive actions such as jumping [42] and, therefore, its occurrence after basketball matchplay is plausible. What is less clear is the reason why the 10:100 Hz ratio was only reduced immediately after the match and not 24 or 48 h later, when low-frequency fatigue may last for several days, at least when it is induced by unaccustomed eccentric contractions [43]. While no additional data or a sound physiological explanation can be provided for the lack of changes in 10:100 Hz ratio after 24 hours, it should be considered that the methodology applied to determine the presence of low-frequency fatigue (i.e. ratio calculated via doublet stimuli) can possibly underestimate the real amount of this type of fatigue compared to other methodologies (i.e., tetanic ratio) [44].

The present study was the first to investigate the time course of muscle fatigue recovery after a simulated basketball match-play. We have shown that baseline values of knee extensor neuromuscular function are restored within 24 h post-match. These findings suggest that basketball players may successfully undergo match-congested periods (i.e. two close matches over a 24–48 h period) without being limited by muscle fatigue. These findings are also supported by the players’ perceived recovery (i.e., TQR), which was unaltered during the days after the simulated basketball match-play, thus further confirming that a 24-h period was acceptable to ensure a sufficient recovery. Therefore, the main limiting factors of basketball matches may be represented by a combination of metabolic mechanisms (e.g., Blact), with minimal muscle damage – potentially related to low-frequency fatigue – that seems to be resolved in 24 h.

When interpreting our findings, some limitations should be considered. First, the outcomes of this study are strongly influenced by the selected simulated basketball match-play protocol, which does not replicate the unpredictable situations, worst case scenarios, psychological stress and technical-tactical tasks faced during an official basketball match. Second, the simulated basketball match-play equates to a playing time of 32 min interspersed with 31 min of passive recovery, and as such it is not necessarily representative of the basketball activity performed by players who experience alternative exposures during matches (e.g., 10 min vs 30 min of match-play). Third, the players recruited for this study were competing in the Spanish 5^th^ division, which may preclude generalizing our findings to players of different sex and age or competing at other levels. Thus, our current findings should be considered as preliminary evidence of muscle fatigue induced by basketball match-play.

### Practical applications

The results of the present study highlight the detrimental effect of basketball activity on knee extensor muscle function, which should be promptly addressed to avoid a reduction in physical performance during matches. Accordingly, players should follow a proper nutritional plan and be sufficiently hydrated pre matches, in addition to the use of supplements that could hinder some of the effects of muscle fatigue (e.g., caffeine or sodium bicarbonate) [8, 45], peripheral fatigue in particular [46]. Beside the general physical condition of players that should be optimized in the days before the match, coaching staff should apply in-game strategies managing players’ load in real-time during match-play (e.g., substitutions, time-out call and tactical decisions – like match ups or type of defence) to limit the effects of muscle fatigue. Although knee extensor muscle function was restored within 24 h post-match in the present study, it is recommended to apply the most appropriate recovery strategies that may help improve readiness and well-being in the days following match-play. We only performed neuromuscular evaluations on the knee extensor muscles, thus future studies should assess matchinduced fatigue on other muscles (e.g., hamstrings, calves) that may experience a different amount and type of fatigue. Also, investigating the time course of muscle fatigue across match-congested periods (e.g., playoff tournament) seems crucial to provide practitioners with complementary information to those presented here (which again are limited to a single simulated basketball match-play).

## CONCLUSIONS

The simulated basketball match-play induced impairments in knee extensor muscle function, with MVC strength (indicative of general muscle fatigue) and electrically-evoked torque responses (indicative of peripheral fatigue) being significantly reduced pre-to-post match and restored within 24 h. Peripheral fatigue affected more the torque evoked at low than high stimulation frequencies, thus highlighting the occurrence of low-frequency fatigue. No signs of post-match central fatigue and variations in perceived recovery at 24 h and 48 h post-match were observed. In conclusion, the present study provided novel and preliminary insights about the etiology and recovery of knee extensors muscle fatigue following simulated basketball match-play, suggesting that a 24-h period seems sufficient to ensure an acceptable recovery among trained players.
